# Assessing the reliability and responsiveness of the SF-6Dv2 and comparing its validity to the EQ-5D-5L among colorectal cancer patients in China

**DOI:** 10.3389/fonc.2025.1657249

**Published:** 2025-09-23

**Authors:** Jiannan Sun, Lijun Xu, Yiyin Cao, Jiaxuan Shi, Yujin Wang, Lemin Wu, Hongjuan Yu, Jiazhuo Liu, Weidong Huang

**Affiliations:** ^1^ Heilongjiang Institute of Health Care Security, Harbin, China; ^2^ School of Health Management, Harbin Medical University, Harbin, China; ^3^ Department of Hematology, The First Affiliated Hospital of Harbin Medical University, Harbin, China; ^4^ Heilongjiang Provincial Health Management Service Evaluation Center, Harbin, China

**Keywords:** SF-6Dv2, psychometric properties, colorectal cancer, EQ-5D-5L, cost-utility analysis

## Abstract

**Objective:**

This study aimed to evaluate the reliability and responsiveness of the SF-6Dv2, and to provide the first comparative assessment of its validity against the EQ-5D-5L in Chinese patients with colorectal cancer (CRC).

**Methods:**

A cross-sectional survey was conducted between August 2022 and December 2023 in three tertiary hospitals in Harbin, China. Eligible CRC patients completed face-to-face baseline interviews to collect demographics, health behaviors, clinical characteristics, EQ-5D-5L, and SF-6Dv2. Follow-up surveys were administered at 7 days and 3 months to collect self-reported health changes and SF-6Dv2. Ceiling and floor effects were assessed by calculating the proportion of respondents reporting the best and worst possible health states. Convergent validity was assessed using Spearman’s correlation with EQ-5D-5L as the reference. Known-groups validity was examined by comparing utility scores across groups categorized by health behaviors and clinical characteristics, testing effect size (ES) and relative efficiency (RE). Agreement was examined using intraclass correlation coefficients (ICC) and Bland-Altman plot. Test-retest reliability of SF-6Dv2 utility and dimension scores was evaluated using ICC and Gwet’s AC over 7 days. Responsiveness was assessed using standardized response mean (SRM) over 4 months.

**Results:**

Baseline included 287 CRC patients; 131 and 111 completed first and second follow-ups. A higher ceiling effect was observed in EQ-5D-5L than in SF-6Dv2 (16.7% vs 3.1%). The Spearman correlation between EQ-5D-5L and SF-6Dv2 utility scores was 0.716 (dimensions: 0.313-0.675). Utility scores from EQ-5D-5L and SF-6Dv2 showed moderate agreement (ICC = 0.686). SF-6Dv2 showed superior known-groups validity in surgical treatment (RE = 1.796) and ECOG groups (RE = 1.953). SF-6Dv2 demonstrated excellent test-retest reliability for utility scores (ICC = 0.866), with Gwet’s AC across dimensions (0.322-0.669). SF-6Dv2 showed greater responsiveness in the worsened group (SRM = 0.788) compared to the improved group (SRM = 0.687).

**Conclusions:**

SF-6Dv2 showed comparable reliability and responsiveness when used in patients with CRC, out-performing EQ-5D-5L in differentiating clinical known-groups and showing promise for cancer practice and research.

## 1 Introduction

Colorectal cancer (CRC) is among the most prevalent malignancies worldwide, with persistently high incidence and mortality. According to GLOBOCAN 2022, CRC ranks third in cancer incidence and second in cancer-related mortality globally, and is the 16th leading cause of death and disability across all diseases. In 2022, CRC (including anal cancer) accounted for over 1.9 million new cases and 904,000 deaths, representing approximately 10% of the global cancer burden (1). In China, CRC is the second most common malignancy and the fourth leading cause of cancer death (2). Treatment typically involves complex, multimodal strategies—such as surgery, chemotherapy, and radiotherapy—that impose substantial physical and psychological burdens. The high disease burden of CRC not only affects patients and families, but also places considerable pressure on healthcare systems and economic resources.

Health technology assessment (HTA) plays a pivotal role in reducing the financial burden of cancer care by informing evidence-based policy decisions (3). International health authorities and methodological guidelines widely recommend cost-utility analysis (CUA) as the preferred form of economic evaluation within HTA frameworks (4, 5). CUA employs the quality-adjusted life year (QALY) as its primary outcome, a composite measure that integrates both the duration and quality of life. QALYs adjust life years by weighting them with health state utilities, which reflect individuals’ preferences for specific health states. The accurate estimation of health state utilities (HSUs) is critical to ensuring the validity and credibility of CUA results (6).

Among the generic multi-attribute utility instruments (MAUIs) designed to estimate QALYs, the EQ-5D and SF-6D are the most widely used globally and are endorsed by multiple national HTA agencies (3). In China, both instruments are included in the *Chinese Guidelines for Pharmacoeconomic Evaluations (2020 edition)* as the recommended instruments for utility measurement in economic evaluations (7). The EQ-5D has been extensively validated in patients with various types of cancer, including breast, lung, gastric, and head and neck cancers, with its psychometric properties well established across most cancer populations (8–14). Several studies have also confirmed its psychometric properties in patients with CRC (8, 15).

The original version of the SF-6D (SF-6Dv1) was developed based on the 36-item Short-Form Health Survey (SF-36) (16). The most recent version of the SF-6D, the SF-6Dv2, was developed by revising ambiguous distinctions between dimension levels and by harmonizing inconsistencies in the positive and negative wording of the SF-6Dv1 (17–19).The original version, SF-6Dv1, has been extensively used in cancer populations (20–22). Compared with the EQ-5D-5L, it contains more dimensions, enabling a more nuanced description of health states in cancer patients. In particular, its “Vitality” dimension has been recognized as a useful indicator for capturing cancer-relevant health outcomes (23, 24). However, the SF-6Dv1 has notable limitations, including unclear ordering of severity across response levels, inconsistent interpretation of dimension wording, and a relatively high rate of missing responses. These issues prompted the development of the revised SF-6Dv2 to improve clarity, consistency, and overall psychometric performance (18, 25–27). To date, country-specific SF-6Dv2 value sets have been developed in several countries-including Canada, Iran, Japan, Australia, the United Kingdom, and China-based on population preferences. These localized value sets provide more culturally relevant support for health economic evaluations (27–33).

Emerging evidence has examined the psychometric properties of SF-6Dv2 in general populations and patients (26, 34–38). Findings consistently show that EQ-5D-5L tends to exhibit a stronger ceiling effect than SF-6Dv2, while SF-6Dv2 demonstrates good convergent validity and test–retest reliability. Notably, responsiveness has been evaluated in only one study-Ding et al.’s investigation of COVID-19 patients in China-which reported favorable results (34). Evidence on known-group validity remains mixed: Xie et al. found superior discriminatory power of SF-6Dv2 compared to EQ-5D-5L in a general Chinese population (35), while Xu et al. reported better performance of EQ-5D-5L among patients with late-onset Pompe disease (38).

Despite its recent development, studies evaluating SF-6Dv2 in Chinese cancer populations remain limited. Available findings indicate good convergent validity and responsiveness in oncology settings (39–41). However, Zhang et al. reported better test–retest reliability for EQ-5D-5L than SF-6Dv2 in lymphoma patients (40), and Xu et al. observed inferior known-group validity of SF-6Dv2 in survivors of classical Hodgkin lymphoma compared to EQ-5D-5L (39). However, to the best of our knowledge, no studies have evaluated the psychometric properties of the SF-6Dv2 in patients with CRC.

The objective of this study was to assess the measurement properties of the SF-6Dv2 among Chinese patients with CRC, with a particular focus on test-retest reliability, convergent validity, known-group validity, and responsiveness.

## 2 Methods

### 2.1 Study design and population

Between August 2022 and December 2023, a total of 287 patients diagnosed with CRC were consecutively recruited from three tertiary-level hospitals in Harbin, the capital city of Heilongjiang Province, China. The inclusion criteria were as follows: (1) confirmed clinical diagnosis of CRC as recorded in medical charts; (2) aged 18 years or older; and (3) able to read and communicate in Chinese and complete the self-reported questionnaires. Eligible patients were approached during hospitalization, provided written informed consent, and participated in face-to-face interviews conducted by trained interviewers. Social-demographic characteristics were collected, including gender, age, registered residence, marital status, educational status, employment status, and economic pressure. Health behavior information included smoking or alcohol consumption, and frequency of health check-ups. Clinical characteristics including cancer type, stage, treatment modality, and Eastern Cancer Oncology Group (ECOG) performance status-were extracted from patients’ inpatient medical records. Health utility assessments were obtained using the Chinese versions of the SF-6Dv2 and EQ-5D-5L. Within seven days after baseline, participants were re-contacted to determine eligibility for the first follow-up. Respondents were asked about their perceived disease progression using a single-item anchor question: “How is your current disease change status?” with three response options: “improved,” “unchanged,” or “worsened.” Participants who reported their health as “unchanged” were included in the test-retest reliability analysis. Four months after baseline, participants were again contacted for a second follow-up using the same questionnaires. These data were used to evaluate the responsiveness of the SF-6Dv2.

The study protocol was approved by the Ethics Committee of Harbin Medical University (approval number: HMUIRB2023005) and conducted in accordance with the Declaration of Helsinki.

### 2.2 Instruments

#### 2.2.1 EQ-5D-5L

The EQ-5D-5L comprises two components to assess health status on the day of the survey. The first component is a descriptive system with five dimensions: Mobility, Self-care, Usual activities, Pain/discomfort, and Anxiety/depression (42). Each dimension has five response levels ranging from “no problems” to “extreme problems” (43), allowing for 3,125 unique health states. These states can be converted into utility scores using a country-specific value set. In this study, utility values were derived using the Chinese EQ-5D-5L value set developed by Luo et al., with scores ranging from -0.391 (for state 55555) to 1.000 (for state 11111) (44). The second component is a vertical visual analogue scale (EQ-VAS), ranging from 0 (worst imaginable health state) to 100 (best imaginable health state) (45).

#### 2.2.2 SF-6Dv2

The SF-6Dv2 is a revised version of the original SF-6Dv1, derived from 10 items of the SF-36v2, and reflects health status over the preceding four weeks (17). The descriptive system comprises six dimensions: Physical functioning, Role limitations, social Functioning, Pain, Mental health, and Vitality (24). The Pain dimension has six levels, while the remaining dimensions have five levels, allowing for a total of 18,750 distinct health states. Utility scores were generated using the Chinese SF-6Dv2 value set developed by Wu et al., with a score range from -0.277 (for state 555655) to 1.000 (for state 111111) (27).

### 2.3 Statistical analysis

#### 2.3.1 Ceiling and floor effects

By assessing the proportion of respondents at the best and worst health states, we evaluated the extent to which each measure was affected by ceiling and floor effects, as well as their related implications. A ceiling or floor effect was considered to be present if more than 15% of respondents achieved the extreme scores at either end of the scale, which would impair the ability of the corresponding dimension to discriminate between different health states.

#### 2.3.2 Convergent validity

Convergent validity was assessed using Spearman’s rank correlation coefficients, a non-parametric statistic that measures the strength and direction of monotonic associations, between the utility scores and dimensions of the EQ-5D-5L and SF-6Dv2. Correlation strength was interpreted as follows: strong (r > 0.50), moderate (r = 0.35–0.49), weak (r = 0.20–0.34), and poor (r < 0.20) (46). Based on previous literature, we hypothesized strong correlations between the Pain dimensions (both in SF-6Dv2 and EQ-5D-5L), and between Mental health dimensions (both in SF-6Dv2 and EQ-5D-5L) (35).

#### 2.3.3 Known-groups validity

Known-groups validity was assessed by comparing SF-6Dv2 utility scores across subgroups with hypothesized differences based on published evidence. It was expected that patients who (1) smoking or alcohol consumption (47, 48), (2) underwent infrequent health check-ups (49), (3) those in cancer stages III–IV (50), (4) had received surgical treatment (51), (5) had ECOG performance scores ≥1 (52), or (6) had EQ-VAS scores ≤65 (35, 53), would report lower utility scores. For each binary variable (e.g., sex), independent *t*-tests, which compare mean differences between two groups under the assumption of approximate normality, were applied. Discriminative ability was further evaluated using effect size (ES) and relative efficiency (RE). ES, a standardized measure of group differences, was calculated for both EQ-5D-5L and SF-6Dv2 by dividing the mean difference in utility scores between groups by the pooled standard deviation (SD) and interpreted as small (ES < 0.2), moderate (0.2 ≤ ES < 0.5), or large (ES ≥ 0.5) (54, 55). RE, an index of comparative efficiency between instruments, was calculated as the squared *t*-statistic of SF-6Dv2 divided by that of EQ-5D-5L. An RE of 1.0 indicates equal discriminative ability, a value >1 suggests superior discriminative performance of SF-6Dv2, and a value <1 indicates stronger performance of EQ-5D-5L (56).

#### 2.3.4 Agreement

Agreement between the utility values derived from EQ-5D-5L and SF-6Dv2 was assessed using intraclass correlation coefficients (ICC), which quantify the degree of agreement or discrepancy between measurements obtained from different instruments. ICC values were interpreted as low (ICC < 0.40), moderate (0.40 ≤ ICC ≤ 0.75), or high (ICC > 0.75) (57). ICC was calculated using a two-way mixed-effects model based on absolute agreement, which accounts for both systematic differences and random errors between instrument (58). A Bland-Altman plot, which graphically displays the mean difference and limits of agreement, was constructed to visually inspect agreement between the two instruments. Agreement was considered satisfactory if the mean difference was close to zero and most values fell within ±1.96 standard deviations of the mean difference, indicating that differences were largely due to random variation rather than systematic bias (59).

#### 2.3.5 Test-Retest reliability

Data from patients reporting “stable” health status in the first follow-up within 7 days were used to assess the test–retest reliability of the SF-6Dv2, which reflects the stability of repeated measurements under unchanged conditions. Test–retest reliability of utility scores and dimension scores was evaluated using ICC and Gwet’s AC, respectively. ICC, a statistic that quantifies the reproducibility of continuous measurements, was interpreted according to the criteria described previously (57). Gwet’s AC, a chance-corrected agreement coefficient less affected by prevalence and marginal distributions than Cohen’s kappa, was used for categorical responses. For Gwet’s AC, values <0.4 indicate poor reliability, values between 0.4 and 0.75 indicate moderate reliability, and values >0.75 indicate good reliability (60).

#### 2.3.6 Responsiveness

Responsiveness was assessed by categorizing patients who self-reported a change in health status at the second follow-up three months later into an “Improved group” and a “Worsen group.” Responsiveness was assessed by categorizing patients who selfreported a change in health status at the second follow-up four months later into an “Improved group” and a “Worsen group.” Responsiveness was evaluated using standardized response means (SRMs), a distribution-based index that quantifies sensitivity to change by standardizing the mean difference with respect to the variability of change scores. SRMs were calculated as the mean change divided by the standard deviation of the change scores and interpreted as small (0.20 ≤ SRM < 0.50), moderate (0.50 ≤ SRM < 0.80), or large (SRM ≥ 0.80) (61).

All statistical analyses were performed using SPSS version 24.0, STATA version 13.0, and AgreeStat360. A p-value < 0.05 was considered statistically significant.

## 3 Results

### 3.1 Demographic characteristics

**Figure 1:**
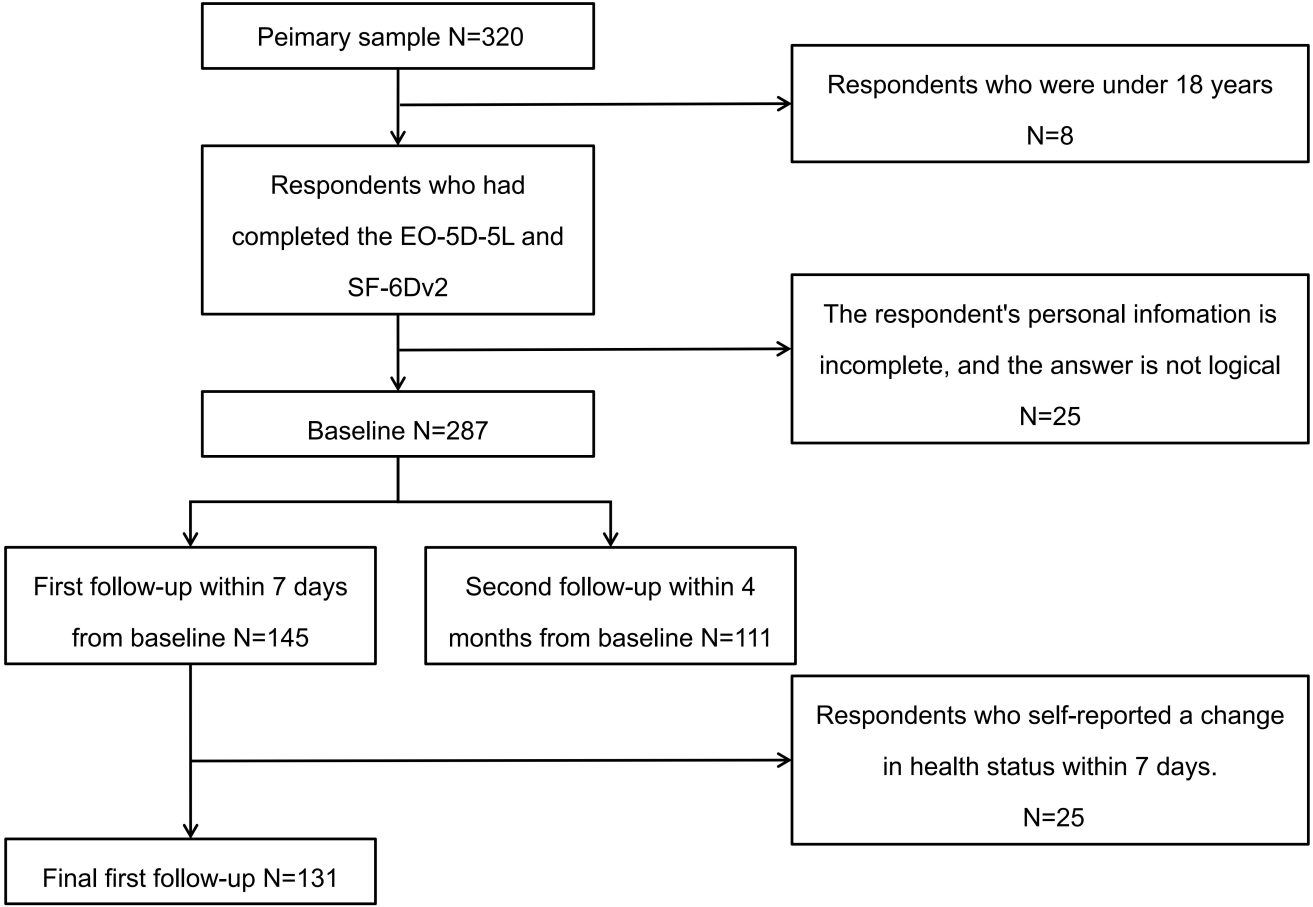
The flowchart of the sample inclusion for the study.

**Figure 1** illustrates the participant flowchart. After excluding individuals who were under 18 years of age, had incomplete responses, or provided logically inconsistent answers, a total of 287 patients with CRC were included at baseline. Among them, 131 patients completed the first follow-up interview and met the criterion of stable health status within seven days, while 111 participants completed the second follow-up interview at four months.

**Table 1** presents the sociodemographic and clinical characteristics of participants across baseline and follow-up assessments. At baseline, 58.5% of the 287 patients were male, with a mean age of 58.14 years. Approximately 69.0% were registered residents of urban areas. Information on patients at the first and second follow-up assessments is presented in **Table 1**.

**Table 1:** Demographic characteristics of patients with colorectal cancer.

Characteristics	Baseline (n=287)	First follow up (n=131)	Second follow up (n=111)
Gender			
Male	168 (58.5%)	66 (50.4%)	63 (56.8%)
Female	119 (41.5%)	65 (49.6%)	48 (43.2%)
Age (Mean)	58.14	56.30	55.87
Registered residence			
City	198 (69.0%)	88 (67.2%)	82 (73.9%)
Countryside	89 (31.0%)	43 (32.8%)	29 (26.1%)
Smoking or alcohol consumption			
Neither smoking nor drinking	172 (59.9%)	76 (58.0%)	69 (62.2%)
Smoking only, not drinking	25 (8.7%)	15 (11.5%)	9 (8.1%)
Drinking only, not smoking	38 (13.2%)	20 (15.3%)	13 (11.7%)
Both smoking and drinking	52 (18.1%)	20 (15.3%)	20 (18.0%)
Frequency of health check-ups			
Regular medical check-ups	100 (34.8%)	46 (35.1%)	30 (27.0%)
Occasional health checkups	97 (33.8%)	44 (33.6%)	48 (43.2%)
Almost never undergo health checkups	90 (31.4%)	41 (31.3%)	33 (29.7%)
Cancer stage			
I	121 (42.2%)	64 (48.9%)	41 (36.9%)
II	57 (19.9%)	23 (17.6%)	14 (12.6%)
III	73 (25.4%)	31 (23.7%)	31 (27.9%)
IV	36 (12.5%)	13 (9.9%)	25 (22.5%)
History of prior CRC treatments			
Surgical treatment	209 (72.8%)	96 (73.3%)	69 (62.2%)
Radiotherapy, chemotherapy	120 (41.8%)	48 (36.6%)	55 (49.5%)
Endocrine therapy	3 (1.0%)	1 (0.8%)	1 (0.9%)
Targeted therapy	43 (15.0%)	18 (13.7%)	30 (27.0%)
TCM Assisted Treatment	23 (8.0%)	4 (3.1%)	8 (7.2%)
other	5 (1.7%)	1 (0.8%)	2 (1.8%)
ECOG			
0	65 (22.6%)	31 (23.7%)	16 (14.4%)
1	122 (42.5%)	58 (44.3%)	65 (58.6%)
2	49 (17.1%)	20 (15.3%)	21 (18.9%)
3	36 (12.5%)	14 (10.7%)	6 (5.4%)
4	15 (5.2%)	8 (6.1%)	3 (2.7%)
SF-6Dv2 index	0.587	0.637	0.720

### 3.2 Ceiling and floor effects

**Figure 2:**
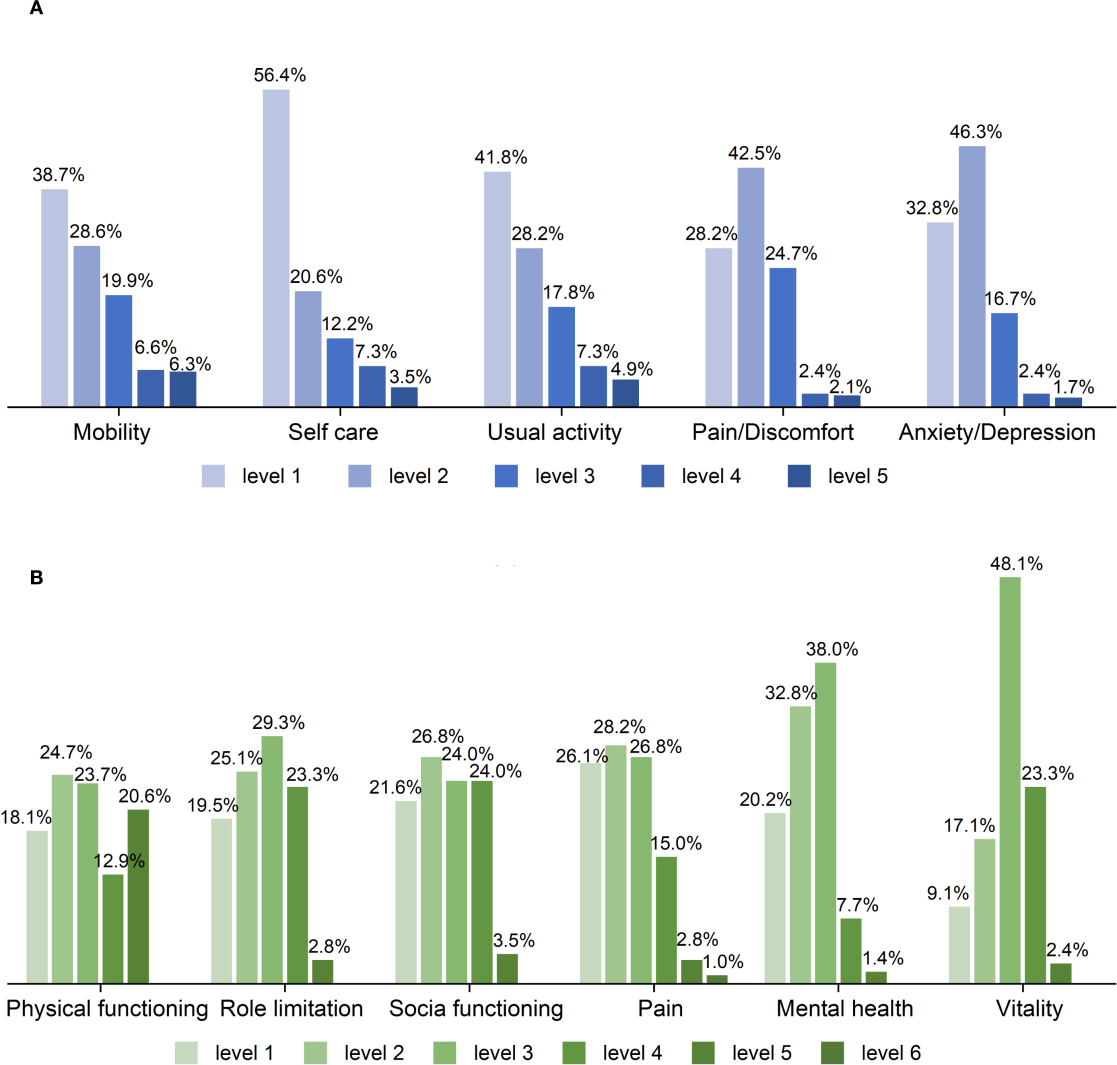
Distribution across levels of the EQ-5D-5L **(A)** and SF-6Dv2 **(B)** dimensions.

As shown in **Figure 2** and **Table A** from **Appendix**, the EQ-5D-5L exhibited a substantial skew towards better health states across dimensions, with a large proportion of respondents reporting “no problems,” particularly in Self-care (56.4%) and Usual activities (41.85%). Notably, 48 patients (16.7%) reported full health (11111). In contrast, the distribution of response levels in the SF-6Dv2 was more balanced, with only 9 patients (3.1%) reporting full health (111111). It is noteworthy that as many as 48.1% of patients reported moderate problems in the Vitality dimension.

### 3.3 Convergent validity

As shown in **Table 2**, the utility scores of SF-6Dv2 and EQ-5D-5L demonstrated a strong correlation (r = 0.716), indicating good convergent validity. At the dimension level, the Physical Functioning dimension of SF-6Dv2 exhibited strong correlations with the Mobility, Self-Care, and Usual Activities dimensions of EQ-5D-5L (r = 0.550, 0.524, and 0.527, respectively). Similarly, the Pain and Mental Health dimensions of SF-6Dv2 were strongly correlated with the Pain/Discomfort and Anxiety/Depression dimensions of EQ-5D-5L (r = 0.675 and 0.627, respectively). In contrast, the Vitality dimension of SF-6Dv2 demonstrated poor correlation with the EQ-5D-5L Pain/discomfort dimension and only moderate correlations with the remaining EQ-5D-5L dimensions.

**Table 2:** Correlation between SF-6Dv2 and EQ-5D-5L dimensions (n=287).

EQ- 5D-5L SF-6Dv2	Mobility	Self-care	Usual activities	Pain/discomfort	Anxiety/depression	EQ-5D-5L index
Physical functioning	0.550	0.524	0.527	0.423	0.410	–
Role limitations	0.453	0.505	0.517	0.459	0.414	–
Social functioning	0.431	0.473	0.515	0.455	0.443	–
Pain	0.497	0.493	0.528	0.675	0.457	–
Mental health	0.487	0.460	0.536	0.471	0.627	–
Vitality	0.361	0.366	0.433	0.313	0.324	–
SF-6Dv2 index	–	–	–	–	–	0.716

Poor (r=0–0.2), weak (r=0.2–0.34), moderate (r=0.35–0.49), strong (r>0.5).

### 3.4 Known-groups validity

As shown in **Table 3**, patients who reported smoking or alcohol consumption, those who underwent infrequent health check-ups., those in cancer stages III–IV, patients who had received surgical treatment, those with ECOG performance scores ≥1, and those with EQ-VAS scores ≤65 had lower mean utility scores on the SF-6Dv2, consistent with the study’s hypotheses. Across all subgroups, mean EQ-5D-5L utility scores were generally higher than those of the SF-6Dv2, with an average RE of 0.876. The SF-6Dv2 demonstrated superior discriminative ability compared to the EQ-5D-5L in differentiating groups by surgical treatment status (ES: 0.366 vs. 0.259, RE >1) and ECOG performance score (ES: 0.651 vs. 0.514, RE >1). Conversely, the EQ-5D-5L exhibited greater discriminative power in distinguishing subgroups by smoking or drinking status (ES: 0.593 vs. 0.299, RE <1), physical examination frequency (ES: 0.661 vs. 0.519, RE <1), cancer stage (ES: 0.317 vs. 0.041, RE <1), and EQ-VAS score category (ES: 0.992 vs. 0.762, RE <1).

**Table 3:** Known-group validity of EQ-5D-5L and SF-6Dv2 (n=287).

Variable	SF-6Dv2				EQ-5D-5L				RE
	Mean (SD)	ES	t	*p*-value	Mean (SD)	ES	t	*p*-value	
Smoked or alcohol consumption									
Yes	0.537 (0.287)	0.299	-2.480	0.014	0.568 (0.367)	0.593	-5.036	0.000	0.243
No	0.620 (0.268)				0.754 (0.260)				
Frequency of health check-ups.									
Yes	0.676 (0.244)	0.519	4.098	0.000	0.802 (0.225)	0.661	4.937	0.000	0.689
No	0.539 (0.284)				0.614 (0.344)				
Cancer stage									
I-II	0.591 (0.304)	0.041	0.317	0.752	0.718 (0.300)	0.317	2.652	0.008	0.014
III-IV	0.580 (0.231)				0.616 (0.343)				
Surgical treatment									
Yes	0.561 (0.290)	0.366	-2.608	0.010	0.657 (0.321)	0.259	-1.946	0.053	1.796
No	0.656 (0.229)				0.739 (0.312)				
ECOG									
0	0.719 (0.251)	0.651	4.505	0.000	0.790 (0.218)	0.514	3.224	0.001	1.953
≥1	0.548 (0.274)				0.647 (0.338)				
EQ-VAS									
≤65	0.444 (0.288)	0.762	-6.081	0.000	0.469 (0.350)	0.992	-8.135	0.000	0.559
>65	0.649 (0.250)				0.771 (0.259)				

ES, Effect size; RE, Relative efficiency; SD, Standard deviations; t, t-statistics.

In the RE calculation, the numerator is the squared t-statistic of SF-6Dv2, and the denominator is the squared t-statistic of EQ-5D-5L. A RE value of 1.0 indicates that SF-6Dv2 has the same discriminative ability as EQ-5D-5L in detecting differences. A RE value greater than 1 suggests that SF-6Dv2 has stronger discriminative ability than EQ-5D-5L, whereas a value less than 1 indicates the opposite. while all other analyses were based on the entire sample (N = 287).

### 3.5 Agreement

The utility scores derived from EQ-5D-5L and SF-6Dv2 demonstrated moderate agreement (ICC = 0.686). As shown in **Appendix Figure B**, Bland–Altman analysis showed that 4.18% of points lay outside the limits of agreement, with over 95% falling within the range of -0.349 to 0.534.

### 3.6 Test-retest reliability

**Table 4** summarizes the test–retest reliability results based on 131 participants who reported no change in health status during the 7-day follow-up period. The ICC for SF-6Dv2 utility scores was 0.866, indicating good reliability. Among individual dimensions, the Physical functioning dimension showed the highest reliability (Gwet’s AC = 0.669), while the Pain dimension exhibited the lowest reliability (Gwet’s AC = 0.322).

**Table 4:** Test-retest reliability of the SF-6Dv2 (n=131).

Dimension	Gwet’s AC	95% CI
Physical functioning	0.669	0.574-0.765
Role limitations	0.512	0.408-0.617
Social functioning	0.655	0.559-0.750
Pain	0.322	0.222-0.422
Mental health	0.639	0.543-0.735
Vitality	0.348	0.242-0.453
SF-6Dv2 index	ICC = 0.866	

Gwet’s AC and ICC: poor (r<0.4), moderate (r=0.41–0.75), strong (r>0.75).

### 3.7 Responsiveness

Among patients who participated in the second follow-up at four months, they were classified into the improved group (n = 27) and the worsened group (n = 36) based on changes in ECOG scores. Responsiveness of SF-6Dv2 utility scores was subsequently evaluated in these patients. Overall, SF-6Dv2 demonstrated higher responsiveness in the worsened group (SRM = 0.788) compared with the improved group (SRM = 0.687). Detailed results are presented in **Table 5**.

**Table 5:** SF-6Dv2 responsiveness in improved and worsened groups (n=63).

Variable	Improved (n=27)	Worsened (n=36)
Baseline (Mean ± SD)	0.501 (0.214)	0.739 (0.129)
Follow-up (Mean + SD)	0.716 (0.184)	0.620 (0.211)
SRM	0.687	-0.788
*p*-value	0.002	0.000

SRM, standardized response means; small effect (0.2-0.5), moderate effect (0.5-0.8), large effect (>0.8); Follow-up: Patients whose ECOG scores changed at the second follow-up survey after 3 months (N = 63).

## 4 Discussion

To the best of our knowledge, this is the first study to systematically evaluate the measurement properties of the SF-6Dv2 in patients with CRC. We found that EQ-5D-5L produced significantly higher utility values and a more pronounced ceiling effect compared to SF-6Dv2, consistent with findings in hemophilia, lymphoma, and general population samples (23, 61, 62). Several factors may explain these differences. First, SF-6Dv2 includes an additional Vitality dimension, which specifically captures cancer-related fatigue and energy loss—common but often underrecognized symptoms that are particularly prevalent among cancer patients (16). Second, SF-6Dv2 uses up to six response levels in dimensions like Pain, improving sensitivity to subtle health changes. Third, the instruments differ in recall period: EQ-5D-5L captures health status “today,” while SF-6Dv2 spans the “past four weeks,” enabling it to report more health issues, especially chronic or fluctuating symptoms, rather than only those present on the assessment day (63).

This study found that the utility values of SF-6Dv2 and EQ-5D-5L showed moderate to high correlation (r=0.716), with relatively high correlation coefficients (r>0.6) in corresponding dimensions such as Pain and Mental health, which is consistent with previous findings (34–38, 40). However, the Vitality dimension of SF-6Dv2 showed weak correlations with all EQ-5D-5L dimensions, likely reflecting fundamental differences in construct and focus. Vitality captures patients’ subjective energy levels and is highly influenced by emotional states (e.g., anxiety, depression) and treatment side effects (e.g., chemotherapy-induced fatigue), resulting in greater variability compared to the more stable, function-based dimensions like Mobility and Usual Activities in EQ-5D-5L. These differences highlight the need to consider measurement heterogeneity when selecting or combining these instruments.

The known-group validity analysis revealed that SF-6Dv2 and EQ-5D-5L exhibited complementary but distinct discriminative strengths. SF-6Dv2 performed better in functional and recovery-related domains, with larger effect sizes and higher relative efficiency for ECOG performance (RE = 1.953) and surgical treatment (RE = 1.796). This advantage likely reflects its multidimensional structure, particularly the “Role Limitation” and “Vitality” domains, together with its 4-week recall period, which allows for capturing sustained impairments, fatigue, and postoperative recovery trajectories beyond short-term fluctuations. Such features make SF-6Dv2 particularly suited to evaluate long-term functional outcomes in CRC patients (64). By contrast, EQ-5D-5L demonstrated stronger sensitivity in lifestyle- and perception-related subgroups. It more clearly distinguished patients by smoking and alcohol consumption (RE = 0.243), cancer stage categories (RE = 0.014), frequency of health check-ups (RE = 0.689), and self-rated health (EQ-VAS, RE = 0.559). These findings underscore the strength of EQ-5D-5L as a concise and efficient tool that effectively reflects lifestyle behaviors, disease burden, preventive health use, and overall health perception (39). Taken together, the two instruments provide complementary perspectives: SF-6Dv2 emphasizes vitality and functional recovery within a longer recall window, while EQ-5D-5L offers a parsimonious yet powerful assessment of lifestyle-related differences and general health status. Their combined use can enrich the evaluation of patient-reported outcomes in CRC patients and support more comprehensive clinical and policy decision-making.

The present study demonstrated good test–retest reliability of SF-6Dv2 utility values (ICC = 0.866). Functional and psychological domains exhibited higher stability, whereas symptom-related domains such as pain and vitality showed lower stability, a pattern likely attributable to the inherently greater short-term variability of symptom states influenced by treatment side effects and emotional fluctuations. Evidence from China further supports our findings: Xie et al. reported excellent test–retest reliability of the SF-6Dv2 in overweight and obese populations (ICC = 0.972) (36). Beyond the Chinese context, Nahvijou et al. observed acceptable test–retest reliability of the SF-6Dv2 among Iranian breast cancer patients (ICC = 0.66) (41). Collectively, these results suggest that the SF-6Dv2 generally demonstrates satisfactory to excellent test–retest reliability across diverse populations, although the magnitude of reliability may vary by disease profile and symptom burden.

This study found that the utility value agreement (ICC = 0.686) between SF-6Dv2 and EQ-5D-5L was higher than that in hemophilia patients (ICC = 0.41) (65, 66) but lower than that in the general population (ICC = 0.78) (67). Bland-Altman analysis showed that the worse the health status, the greater the difference in utility values between the two instruments, which was consistent with the findings in lymphoma patients (68).

This study demonstrated that the SF-6Dv2 was sensitive to health status changes in CRC, with greater responsiveness observed in the worsened group than in the improved group. The larger utility declines among deteriorating patients suggest an asymmetric perception of health changes over the disease course. In our cohort, in which more than half of the patients underwent surgical treatment, tumor resection was likely the principal determinant of utility gains; however, recovery trajectories were frequently constrained by enduring sequelae (e.g., stoma-related complications, bowel dysfunction) and persistent psychological distress (e.g., fear of recurrence), which attenuated perceived improvement and limited responsiveness in the improved group (69). In contrast, evidence from hematologic malignancies—where EQ-5D-5L, SF-6Dv2, and QLU-C10D were employed—has indicated stronger responsiveness in improved rather than worsened patients (40). These divergent patterns underscore cancer-type differences in the salience and appraisal of health transitions: in CRC, deterioration tends to be immediate and salient, whereas improvement, even post-resection, is experienced as gradual and incomplete. Collectively, our findings affirm the capacity of SF-6Dv2 to capture clinically meaningful change, while emphasizing the importance of interpreting responsiveness within the context of disease trajectory and patient-reported experience.

This study has several limitations. First, the use of convenience sampling with voluntary participation may have introduced selection bias, as participants were likely to have milder conditions or better treatment responses. This could lead to an underestimation of disease burden and reduce the ability to detect differences in validity across health status subgroups, thereby limiting the assessment of SF-6Dv2’s sensitivity. Second, EQ-5D-5L data were not collected simultaneously during the test-retest period. Although the reliability of SF-6Dv2 was assessed through repeated measurements, the lack of a comparator restricted the evaluation of longitudinal consistency between instruments, limiting conclusions regarding SF-6Dv2’s suitability for monitoring disease progression. Future studies should use nationally representative, stratified, multi-center samples to enhance generalizability, and include cancer-specific instruments (e.g., EORTC QLQ-C30) for criterion validation. Such approaches would allow a more comprehensive assessment of SF-6Dv2’s construct validity, responsiveness, and cross-instrument consistency, clarifying its applicability and potential for optimization in oncology-related economic and clinical research.

## 5 Conclusion

To the best of our knowledge, this is the first study to systematically evaluate the measurement properties of the SF-6Dv2 in patients with CRC. SF-6Dv2 showed comparable reliability and responsiveness when used in patients with CRC, out-performing EQ-5D-5L in differentiating clinical known-groups and showing promise for cancer practice and research.

## Data Availability

The raw data supporting the conclusions of this article will be made available by the authors, without undue reservation.
