# Supplementary material for: Assessing the reliability and responsiveness of the SF-6Dv2 and comparing its validity to the EQ-5D-5L among colorectal cancer patients in China
Source: Front Oncol. 2025 Sep 23;15:1657249. doi: 10.3389/fonc.2025.1657249 (PMC12500642; doi:10.3389/fonc.2025.1657249)
Supplement: Supplementary file 1 [file DataSheet1.docx]

Supplementary Material

# Appendix

**Table A** The baseline response distributions of SF-6Dv2 and EQ-5D-5L

| EQ-5D-5L | | | SF-6Dv2 | | |
| --- | --- | --- | --- | --- | --- |
| Dimension | Level | n (%) | Dimension | Level | n (%) |
| Mobility | 1 | 111 (38.7%) | Physical functioning | 1 | 52 (18.1%) |
|  | 2 | 82 (28.6%) |  | 2 | 71 (24.7%) |
|  | 3 | 57 (19.9%) |  | 3 | 68 (23.7%) |
|  | 4 | 19 (6.6%) |  | 4 | 37 (12.9%) |
|  | 5 | 18 (6.3%) |  | 5 | 59 (20.6%) |
| Self-care | 1 | 162 (56.4%) | Role limitations | 1 | 56 (19.5%) |
|  | 2 | 59 (20.6%) |  | 2 | 72 (25.1%) |
|  | 3 | 35 (12.2%) |  | 3 | 84 (29.3%) |
|  | 4 | 21 (7.3%) |  | 4 | 67 (23.3%) |
|  | 5 | 10 (3.5%) |  | 5 | 8 (2.8%) |
| Usual activities | 1 | 120 (41.8%) | Social functioning | 1 | 62 (21.6%) |
|  | 2 | 81 (28.2%) |  | 2 | 77 (26.8%) |
|  | 3 | 51 (17.8%) |  | 3 | 69 (24.0%) |
|  | 4 | 21 (7.3%) |  | 4 | 69 (24.0%) |
|  | 5 | 14 (4.9%) |  | 5 | 10 (3.5%) |
| Pain/discomfort | 1 | 81 (28.2%) | Pain | 1 | 75 (26.1%) |
|  | 2 | 122 (42.5%) |  | 2 | 81 (28.2%) |
|  | 3 | 71 (24.7%) |  | 3 | 77 (26.8%) |
|  | 4 | 7 (2.4%) |  | 4 | 43 (15.0%) |
|  | 5 | 6 (2.1%) |  | 5 | 8 (2.8%) |
| Anxiety/depression | 1 | 94 (32.8%) |  | 6 | 3 (1.0%) |
|  | 2 | 133 (46.3%) | Mental health | 1 | 58 (20.2%) |
|  | 3 | 48 (16.7%) |  | 2 | 94 (32.8%) |
|  | 4 | 7 (2.4%) |  | 3 | 109 (38.0%) |
|  | 5 | 5 (1.7%) |  | 4 | 22 (7.7%) |
| Ceiling | 11111 | 48 (16.7%) |  | 5 | 4 (1.4%) |
| Floor | 55555 | 1 (0.3%) | Vitality | 1 | 26 (9.1%) |
|  |  |  |  | 2 | 49 (17.1%) |
|  |  |  |  | 3 | 138 (48.1%) |
|  |  |  |  | 4 | 67 (23.3%) |
|  |  |  |  | 5 | 7 (2.4%) |
|  |  |  | Ceiling | 111111 | 9 (3.1%) |
|  |  |  | Floor | 555655 | 0 |


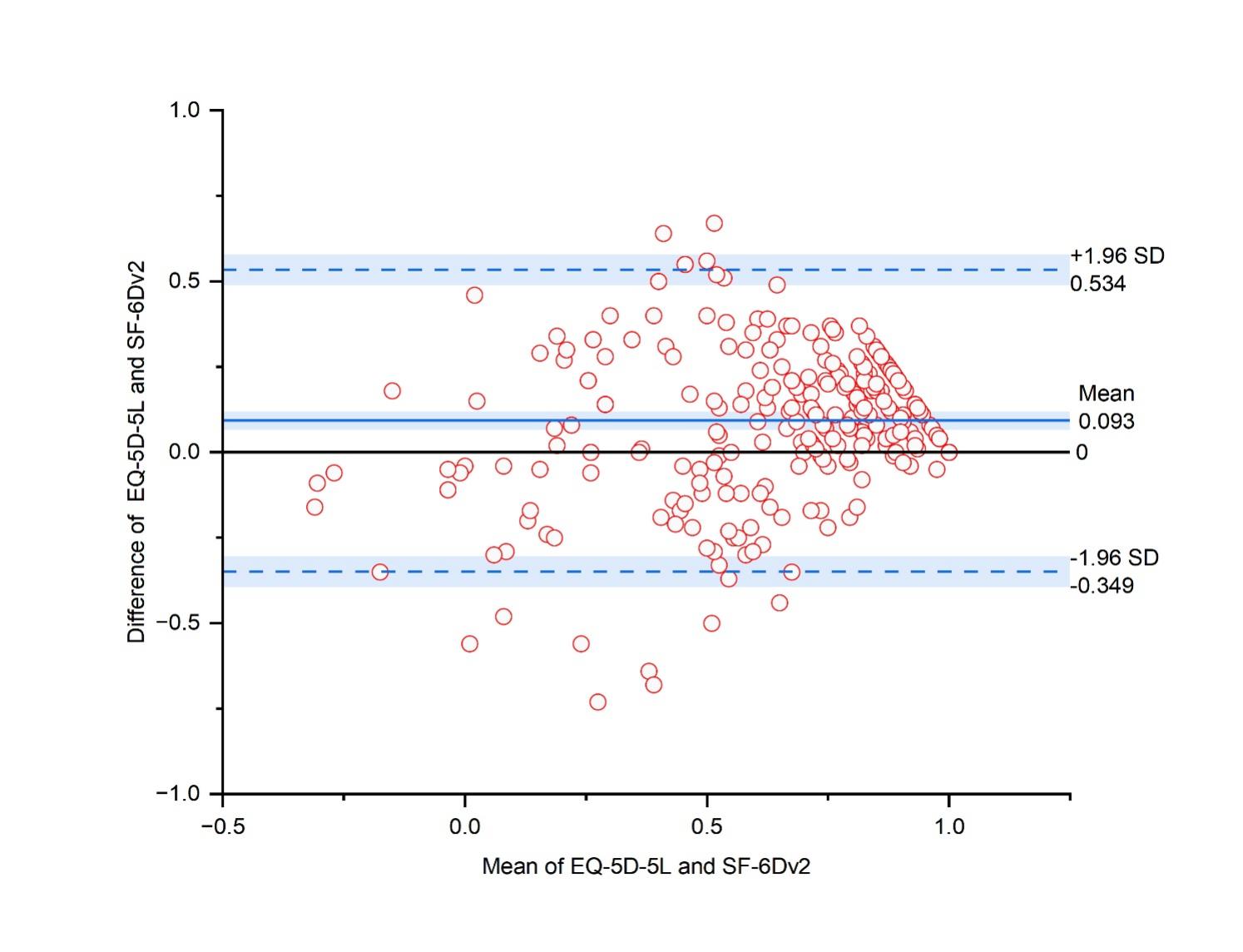


**Fig B.** Bland-Altman plot of EQ-5D-5L and SF-6Dv2 utility scores SD: Standard deviations
